# The evolving species concepts used for yeasts: from phenotypes and genomes to speciation networks

**DOI:** 10.1007/s13225-021-00475-9

**Published:** 2021-06-26

**Authors:** Teun Boekhout, M. Catherine Aime, Dominik Begerow, Toni Gabaldón, Joseph Heitman, Martin Kemler, Kantarawee Khayhan, Marc-André Lachance, Edward J. Louis, Sheng Sun, Duong Vu, Andrey Yurkov

**Affiliations:** 1 Westerdijk Fungal Biodiversity Institute, Utrecht, The Netherlands; 2 Institute of Biodiversity and Ecosystem Dynamics (IBED), University of Amsterdam, Amsterdam, The Netherlands; 3 Dept Botany and Plant Pathology, College of Agriculture, Purdue University, West Lafayette, IN 47907 USA; 4 Evolution of Plants and Fungi, Ruhr-University Bochum, 44801 Bochum, Germany; 5 Barcelona Supercomputing Centre (BSC–CNS), Jordi Girona, 29, 08034 Barcelona, Spain; 6 Institute for Research in Biomedicine (IRB Barcelona), The Barcelona Institute of Science and Technology, Baldiri Reixac, 10, 08028 Barcelona, Spain; 7 Catalan Institution for Research and Advanced Studies (ICREA), Barcelona, Spain; 8 Department of Molecular Genetics and Microbiology, Duke University Medical Center, Durham, NC 27710 USA; 9 Department of Microbiology and Parasitology, Faculty of Medical Sciences, University of Phayao, Phayao, 56000 Thailand; 10 Department of Biology, University of Western Ontario, London, ON N6A 5B7 Canada; 11 Department of Genetics and Genome Biology, Genetic Architecture of Complex Traits, University of Leicester, Leicester, LE1 7RH UK; 12 German Collection of Microorganisms and Cell Cultures, Leibniz Institute DSMZ, Brunswick, Germany

**Keywords:** Fungi, Species concepts, Comparative genomics, Hybrids, Nomenclature, Taxonomy

## Abstract

Here we review how evolving species concepts have been applied to understand yeast diversity. Initially, a phenotypic species concept was utilized taking into consideration morphological aspects of colonies and cells, and growth profiles. Later the biological species concept was added, which applied data from mating experiments. Biophysical measurements of DNA similarity between isolates were an early measure that became more broadly applied with the advent of sequencing technology, leading to a sequence-based species concept using comparisons of parts of the ribosomal DNA. At present phylogenetic species concepts that employ sequence data of rDNA and other genes are universally applied in fungal taxonomy, including yeasts, because various studies revealed a relatively good correlation between the biological species concept and sequence divergence. The application of genome information is becoming increasingly common, and we strongly recommend the use of complete, rather than draft genomes to improve our understanding of species and their genome and genetic dynamics. Complete genomes allow in-depth comparisons on the evolvability of genomes and, consequently, of the species to which they belong. Hybridization seems a relatively common phenomenon and has been observed in all major fungal lineages that contain yeasts. Note that hybrids may greatly differ in their post-hybridization development. Future in-depth studies, initially using some model species or complexes may shift the traditional species concept as isolated clusters of genetically compatible isolates to a cohesive speciation network in which such clusters are interconnected by genetic processes, such as hybridization.

## Introduction

The term ‘species’ is likely one of the most widely applied in biology, but it is also among those most debated. Names of yeast species are extensively used, e.g., to identify a subject analysed in scientific studies, to assess biodiversity and the intrinsic value of nature, to decide on treatment in the case of infections of plants, humans, and other animals, to protect intellectual property, to communicate information on species-specific properties, and to search related information in databases such as GenBank, PubMed or GBIF. Yet, the definition of a ‘species’ is still debated, especially in the areas of evolutionary biology and systematics. Many different species concepts have been applied (Table 1) that are also relevant for microbes, including yeasts (Lücking et al. 2020). Species concepts have evolved over time. In the early era of systematics, species described by John Ray, Carl Linnaeus, and Alphonse de Candolle were considered to be immutable and they used morphological and sexual features for grouping living organisms, especially plants, to place them into taxa, including genera and species (Ray 1686; Linnaeus 1758; De Candolle 1813). Since the publication of ‘*On the origin of species by means of natural selection, or the preservation of favoured races in the struggle for life*’ (Darwin 1859) various modern species concepts have emerged to address limitations of the traditional species definitions (Taylor et al. 2000; Mayden 2002; De Queiroz 2007; Aldhebiani 2018). The use of different species concepts is not a negative notion but is a consequence of an increase of scientific knowledge and the increasing types and number of data available for taxonomists.

**Table 1:** Some species concepts used in the systematics of yeasts (adapted from Taylor et al. 2000; De Queiroz 2007; Aldhebiani 2018)

Species concept	Definition of species	Features used
**Phenotypic**	A set of organisms that look similar to each other and distinct from other such sets	Morphology, physiology, etc.
**Genetic**	Groups of isolates that share > 70% DNA similarity based on DNA reassociation experiments; 0-3 differences in the D1/D2 domains of the Large Subunit ribosomal DNA	DNA reassociation values; # nucleotide differences
**Biological**	Groups of actually or potentially interbreeding natural populations which are reproductively isolated from other such groups	Crosses, fertility
Ecological	A species is a group of organisms that inhabit the same niche or habitat and that is dissimilar from other such species	Ecology, ecophysiology, fitness
Evolutionary	A single lineage of ancestor descendant populations of organisms which maintains its identity from other such lineages and which has its own evolutionary tendencies and historical fate	All features
Cohesion/Genealogical	An evolutionary lineage that serves as the arena of action of basic microevolutionary forces, such as gene flow, genetic drift and natural selection	All features
**Phylogenetic**	A group of organisms that share unique traits, which are distinctive from other such groups, and that form a monophyletic cluster	Mainly nucleotide sequences, genomes
**Genealogical Concordance Phylogenetic Species Recognition**	A group of organisms that form cohesive clades for which gene phylogenies are concordant.	Nucleotide/amino acid sequences, genomes
Consolidated	Polyphasic approach combining aspects of phenotypic, ecological and phylogenetic species concepts	All features

Species concepts widely used for recognizing yeast species are indicated in bold

The conceptual breakthrough in our understanding of species resulted from Charles Darwin’s and Alfred Russel Wallace’s thoughts on species as dynamic entities in nature that evolve over time (Darwin and Wallace 1858; Darwin 1859). This point of view was in stark contrast to the previously held belief in species as static and fixed units designed according to the taste of an almighty divine power. Next to this major philosophical paradigm shift, technological innovations helped to better understand the dynamic nature of species during their evolutionary past, but also in the present. Knowing DNA (and RNA) as the fundamental carrier of biological information, and how it changes over time, due to mutation and selection, is key to understanding mechanisms of speciation.

In this review we will address: (1) the main species concepts that have been applied throughout time in yeast taxonomy; (2) the experimental approaches to test the biological species concept (BSC) and compare this with other species concepts; (3) the role and importance of hybrids; (4) the impact of comparative genomics on understanding yeast species; (5) The role and utility of DNA sequences in the practical recognition of yeast species, including barcode analysis on species identification; and (6) practicalities involved when describing yeast species, including nomenclatural aspects.

**Fig. 1:**
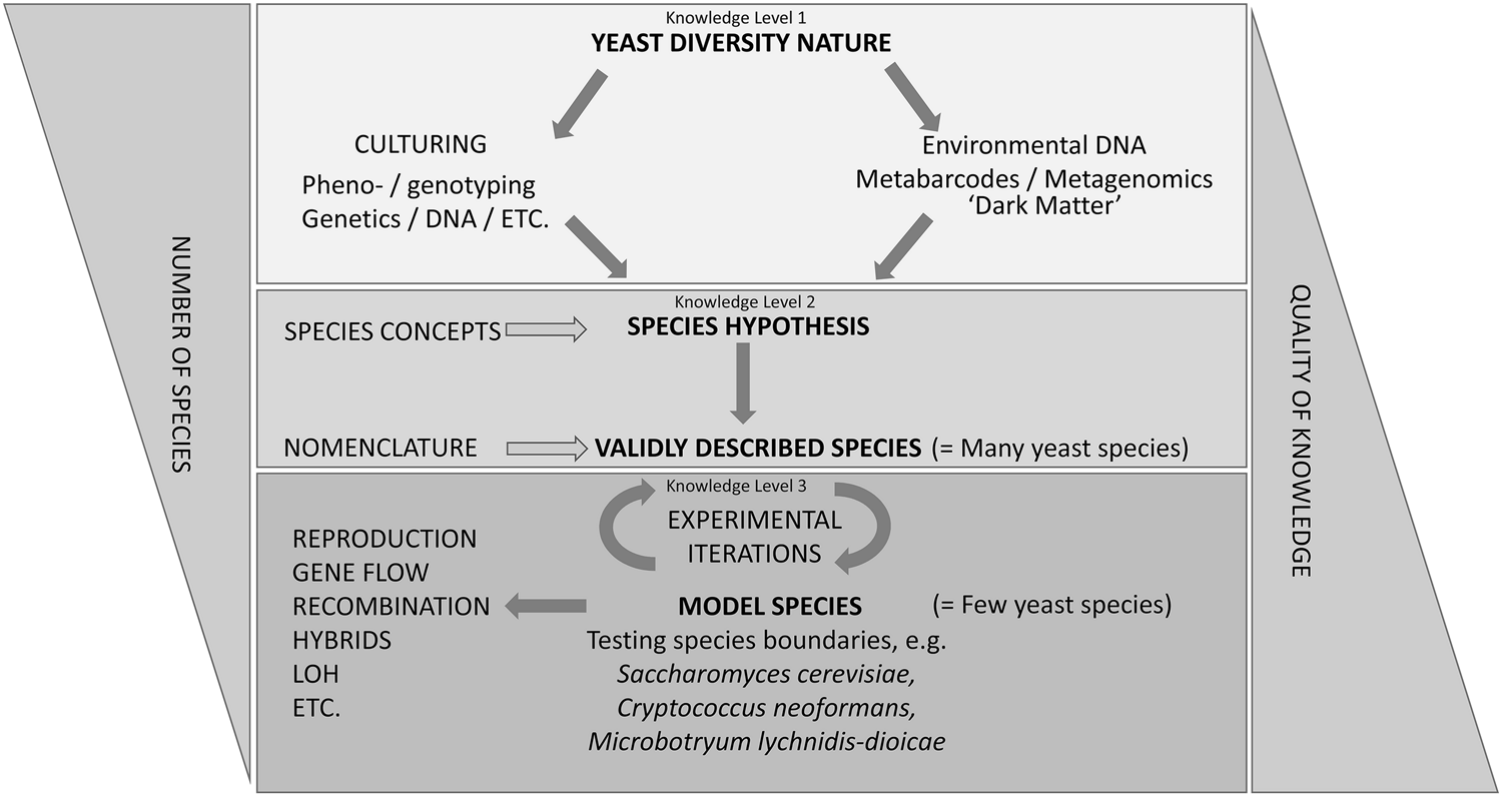
Scheme showing increase of knowledge of yeast biodiversity with three levels of knowledge on certainty of species recognition

As unicellular fungi that are referred to as yeasts are a polyphyletic assemblage that occur in two fungal phyla, Ascomycota (viz. *Saccharomycotina* and *Taphrinomycotina*) and Basidiomycota (viz. *Ustilaginomycotina, Pucciniomycotina*, and *Agaricomycotina*/*Tremellomycetes*), we will present examples from these diverse lineages of fungi. Finally, we present a scheme that recognizes three levels of knowledge with increased confidence for species recognition and takes into account the genetic and evolutionary complexity of species (Fig. 1).

## Species concepts

Systematists have the mandate of discovering and delineating new species, documenting their properties, assigning them correctly to higher taxa, and providing means for accurate identification. These tasks are interrelated but distinct. In its simplest iteration, the description of a yeast species must entail the valid publication of a protologue that specifies a type specimen to which a binomial is permanently attached, as specified by the ‘*International Code of Nomenclature for Algae, Fungi, and Plants’* (ICNafp, Turland et al. 2018). Ideally, a good species description should go well beyond the mere fulfilment of formal requirements of the ICNafp (Aime et al. 2021). Linnaeus (1758) famously stated that the species is the work of nature, while much of everything else in systematics is a human construct. The heart of the species description, therefore, is the demonstration that a strain or collection of strains represent a distinct species, a topic worthy of its own discussion.

Several species concepts have been applied to describe and interpret yeast diversity (Table 1), the most important being: 1. The Phenotypic Species Concept (PhenSC), with its molecular variant, the Genetic Species Concept (GSC). Ecological parameters have been used to recognize ecological species that represents a particular variety of the PhenSC under the so-called Ecological Species Concept (Kudryavtsev 1960; Barnett 2004). Growth properties on e.g. high sugar containing or high salt-contaning media, as well as nutritional growth profiles traditionally used to characterize yeast species can be seen as such ecological parameters, and the same holds for speciation due to host specificity; 2. The Biological Species Concept (BSC); and 3. The Phylogenetic Species Concept (PSC) with its operational version, the Genealogical Concordance Phylogenetic Species recognition (GCPSR). Recently, also the Consolidated Species Concept has been proposed that uses a polyphasic approach combining aspects of the PhenSC and PSC (Hawksworth and Lücking 2017; Quaedvlieg et al. 2014). This seems a pragmatic species concept that integrates various kinds of data into an operational species. It has also been emphasized that species boundaries are porous/semipermeable, at least under certain conditions (Hausdorf 2011; Steenkamp et al. 2017). This porosity may be due to cross-species gene flow between recently diverged species or closely related allopatric species and may confer a selective advantage. The authors listed hybridisation, introgressive hybridisation and horizontal gene flow as a consequence of this porosity/semipermeability (Steenkamp et al. 2017). Recently, the Differential Fitness Species Concept (DFSC) has been proposed as “groups of individuals that are reciprocally characterized by features that would have negative fitness effects in other groups and that cannot be regularly exchanged between groups upon contact” (Hausdorf 2011). This species concept considers all genetic changes that confer differences in fitness and differs from the BSC in that the exchange of species-specific features is not only restricted by reproductive isolation, but also by selection (Hausdorf 2011). As far as we know the DFSC has not yet been applied to fungi, but it is likely that some model species studied in speciation research, such as *Saccharomyces cerevisiae*, will be useful to test its relevance. Very recently, it has been proposed that variation in homoplasy of homologous DNA sequences when compared within and between species can be used as an additional feature to recognize species (Conti et al. 2021). Examples came from the genera *Candida, Debaryomyces, Kazachstania* and *Saccharomyces* (Conti et al. 2021).

For yeasts, the PhenSC was widely used before the introduction of molecular data (Barnett 2004; Giraud et al. 2008; Taylor et al. 2000). Yeast species have been recognized for decades based on phenotypic properties, including colony and cellular morphologies, growth requirements using arrays of different carbon and nitrogen sources, fermentation of various sugars, growth at different temperatures, starch formation, or gelatine liquefaction (Kurtzman et al. 2011b).

Features of DNA were introduced in the 1970s as quantitative criteria to distinguish and compare yeast species when Phaff and co-workers initiated the analysis of the guanine and cytosine content (mol% G+C) and DNA reassociation studies. From these studies it emerged that strains differing by 1–2 mol % G+C likely represented different species (Martini and Phaff 1973; Price et al. 1978; reviewed in Kurtzman et al. 2011c; Lachance 2018). Along similar lines, yeast strains differing by a ≤ 80% DNA reassociation value (later lowered to 70%) were considered to be conspecific, and values ranging between 40 and 70% were considered to indicate varieties or subspecies (Kurtzman 1987, 1998). In the early 1990s sequence analysis of parts of the ribosomal RNA (rRNA) gene, also referred to as ribosomal DNA (rDNA), was introduced (e.g., Peterson and Kurtzman 1991). These authors studied the D2 domain of the Large Subunit (LSU) rRNA and suggested that conspecific strains showed < 1% nucleotide divergence. Kurtzman and Robnett (1998) studied sequence variation in the D1/D2 domains of the LSU rDNA of ca. 500 species of ascomycetous yeasts and compared the application of the Biological Species Concept (BSC, see below) with the amount of sequence divergence present in the LSU rDNA D1/D2 domains. In the chapter ‘*Gene sequence analyses and other DNA*-*based methods for yeast species recognition’* published in the 5th edition of ‘*The Yeasts, a Taxonomic Study’* (‘*The Yeasts’*) the main findings were summarized as follows: conspecifics may differ by up to three nucleotide substitutions and species differ with six or more substitutions of the approximate 600 nucleotides of the D1/D2 domains of the LSU rDNA (Kurtzman et al. 2011c). This of course leaves room for the interpretation of strains that differ with four or five nucleotides. Similar data were obtained by Fell et al. (2000) for basidiomycetous yeasts and these authors stated that conspecific strains had ≤ 3 nucleotides and different species ≥ 6 nucleotides in the D1/D2 domains. This interpretation leaves a gap for the range of 4–6 nucleotide differences in the D1/D2 domains of the LSU rDNA. In a follow-up study including both D1/D2 domains and the Internal Transcribed Spacer (ITS) 1 and 2, including the 5.8S rRNA gene, Scorzetti et al. (2002) came to different conclusions. They wrote “a boundary between species (a prerequisite number of nucleotide differences in either region to separate species) was not defined by our study. The answer undoubtedly lies not in the number of differences, but rather in the significance and function of the site mutations.” In Table 10.1 of the 5th edition of *The Yeasts*, however, the following values were presented:  % nDNA relatedness 70–100% correlated with 0–3 nucleotide differences in the D1/D2 domains of LSU rDNA implying conspecificity; 0–20% nDNA relatedness correlated with 6–250 nucleotide differences in the D1/D2 domains of LSU rDNA indicating different species (Kurtzman et al. 2011c). The above distance measures have been used by the late Cletus P. Kurtzman to introduce a Genetic Species Concept (GSC) that circumscribes yeast species based on a measure of genetic distance obtained indirectly from DNA reassociation kinetics or directly from the comparison of aligned sequences. Thus, the GSC holds a position intermediate to the Phenotypic-, Biological-, and Phylogenetic species concepts, and is best interpreted as an operational tool to detect and circumscribe potential new species. However, it is fair to state that values such as the number of nucleotide differences that predict species boundaries should only be used as a first approximation of what might represent actual yeast species. Lachance (2018) emphasized the lack of a thorough statistical analysis of such threshold values. In addition, some notable exceptions to this rule of thumb have been observed, e.g., the D2 domain of mating strains of *Clavispora lusitaniae* showed up to 32 nucleotide substitutions (Lachance et al. 2003).

In addition to the D1/D2 domains of LSU rDNA, other regions of the rRNA gene cluster have been used, e.g., the Small Subunit (SSU) rRNA gene, the ITS 1 + 2, and the Intergenic Spacer (IGS). Yeast taxonomists have also used partial sequences of protein-coding genes such as the Translation Elongation Factor 1α (*TEF1*) and RNA- and DNA-directed RNA polymerase II subunits (*RPB1* and *RBP*2). The phylogenetic interpretation of such sequence data has led to the use of the Phylogenetic Species Concept (PSC) in yeasts. In most cases, however, phylogenetic analyses have taken a secondary role, as sequence differences have been interpreted as distance measures for an operational species recognition using the GSC. Taylor and collaborators presented a seminal paper on species concepts in use in mycology (Taylor et al. 2000). These authors proposed the Genealogical Concordance Phylogenetic Species Recognition (GCPSR), which compares genealogies resulting from several unrelated genes to infer species boundaries for fungi. Thus, the generation of multiple unlinked gene sequences is required for application of the GCPSR; see, e.g., Hagen et al. (2015). The analysis of multiple loci (a.k.a. Multi-Locus Sequence Analysis, MLSA) is likely a transition towards the use of fragmented or, preferably, entire genomes (see below) for species recognition.

## The biological species concept (BSC)

The BSC holds a central position in eukaryote biology and thus also in mycology, as it is based on the estimation of gene flow among and between populations that may, or may not, belong to different species. Full application of the BSC is in principle limited to organisms that depend on sexual reproduction for their reproductive success. *Prima facie,* yeasts would fare poorly in this context, as they propagate primarily by mitotic division. However, the prolificity of a species extends well beyond numbers, and the greatest benefit of sexual reproduction lies not in propagation, but in the fitness benefits accrued through genetic recombination (McDonald et al. 2016). A large proportion (45%) of ascomycetous yeast species are known exclusively from their asexual state (Kurtzman et al. 2011c), although many apparently asexual species may in fact be known from a single haploid mating type due to insufficient sampling. In many cases, this question can be answered by the characterization of mating genes in their genomes (Yurkov et al. 2015; Kijpornyongpan et al. 2018; Krassowski et al. 2019; Passer et al. 2019). Below we will explore the impact of the BSC in various fungal lineages to which yeasts belong.

### Lessons from *Saccharomyces*, *Saccharomycotina*, *Ascomycota*

The biological species concept has been used in *Saccharomyces* species for decades, well before genome sequences became available and before rRNA gene and ITS sequences were used routinely (Naumov 1987). Most wild isolates of *Saccharomyces* spp. [also referred to simply as ‘yeast’ or ‘budding yeast’ in many publications] are homothallic, due to the action of the HO endonuclease on the mating type locus, resulting in haploid mother cells switching mating type and forming diploid zygotes with daughter cells or undergoing mating between sister spores due to proximity (see for example Greig 2009; Ono et al. 2020). Despite this, mating between different isolates can be accomplished using spore-to-spore mating (Naumov 1987) which can be enhanced with auxotrophic markers, allowing selection and confirmation of zygotes. Using tester strains from known species, crosses to strains of unknown lineage result in viable spores at reasonable frequencies (> 50%) in conspecifics (same species), or very low spore viability (~ 1% or less), between different species, due to chromosomal abnormalities or genetic problems leading to infertility. Several new species were identified solely on this basis (Naumov et al. 1995a, b), and their boundaries were subsequently supported by ITS sequence comparisons (Naumov et al. 2000). Whole genome sequences further refine species definitions by identifying hybrids, introgression, or cases of reproductive isolation due to Gross Chromosomal Rearrangements (GCRs) without notable sequence divergence, as is the case with *S. cariocanus*, now considered part of the American population of *S. paradoxus* but differing by four reciprocal translocations. Eight *Saccharomyces* species are now recognised, defined by a combination of the BSC and whole genome sequence comparisons (Dujon and Louis 2017; Ono et al. 2020).

Post-zygotic reproductive isolation can be caused by several mechanisms. GCRs can lead to unbalanced chromosomal segments and lethality as is the case with *S. cariocanus*, whose four reciprocal translocations theoretically reduce spore viability in heterozygotes to around 6%, close to the observed value (Liti et al. 2006; Dujon and Louis 2017). Other examples are found, such as the Malaysian population of *S. cerevisiae* (Liti et al. 2009) and other *S. cerevisiae* strains surveyed (Hou et al. 2014). Although GCRs do exist between many of the species, the genome of some distinct species are colinear, as for example *S. cerevisiae* and *S. paradoxus*, such that GCRs do not explain their reproductive isolation. A second mechanism involves Bateson–Dobshansky–Muller incompatibilities where genetic interactions cause sterility. There are certainly a few well-characterised examples of existing incompatibilities, particularly those observed between the nuclear and mitochondrial genomes (Lee et al. 2008), but these have been difficult to find, and it is not clear that they occurred prior to speciation (Louis 2009, 2011). The third mechanism is sequence divergence acted upon by the mismatch repair system during meiosis (Chambers et al. 1996; Hunter et al. 1996; Greig et al. 2003), where recombination intermediates are aborted resulting in lack of essential crossovers between homeologues required for proper chromosome segregation.

### Lessons from other *Saccharomycotina*, *Ascomycota*

Among yeasts with a demonstrated sexual cycle, only 24% have been shown to have fixed mating types (heterothallic) that would ensure that a sexual cycle has the potential to allow full participation in a common gene pool. In fact, a substantial proportion of heterothallic yeast species exist in nature as stable diploids whose meiotic division leads to the formation of short-lived ascospores that engage in sister-pair matings soon after gemination. In such cases, the potential for recombination may not differ much from that of strictly asexual species. Yeasts epitomize the paradox of sex. Speijer et al. (2015) argue that sexual reproduction lies at the very essence of what it is to be an eukaryote. Yet, yeasts have repeatedly evolved multiple, elegant mechanisms that enable continuous mating-type switching, which would tend to reduce genetic diversity in a population instead of fostering it. An analysis of 332 genomes (Krassowski et al. 2019) found that 42% of the sexual species studied at the genome level exhibit one of five forms of homothallism, which allows sexual reproduction within an essentially clonal population. In haplontic species, this can be accomplished by mating type switching within a population, which allows the conjugation of sister cells, as in *Zygosaccharomyces* species, or by fusion of sister nuclei, one of which returns to its mother cell from an aborted bud, as typified by *Debaryomyces*, *Torulaspora*, or *Nadsonia* species (Kurtzman et al. 2011c). The possibility exists that ascosporulation in such cases has been co-opted for dormancy or increased resistance to environmental stress (e.g., passage through insects guts). These mechanisms do not entirely preclude outcrossing but make it difficult to use the BSC as a criterion for species recognition. For example, the isolation of recombinants in hybrid crosses caused van der Walt and Johannsen (1984) to reduce several species of *Kluyveromyces* to varieties of *K. marxianus*, but later studies based primarily on genetic divergence (Lachance 2011) provided evidence that assignment to three distinct species, *K.* *marxianus*, *K. lactis*, and *K. dobzhanskii*, was a better reflection of biology. Naumov et al. (2016) found a good correspondence between hybrid fertility and phylogenetic relatedness in species of the homothallic genus *Komagataella*. Interestingly, the genus was used by Naumov (2015) as an example of a “genetic genus”, where prezygotic isolation is used to delineate genera, a concept that he extended also to *Saccharomyces*, *Kluyveromyces*, *Arthroascus* (a.k.a. *Saccharomycopsis*), *Galactomyces*, *Metschnikowia*, *Phaffomyces*, *Williopsis*, and *Zygowilliopsis* (the last two are currently assigned to *Cyberlindnera* and *Barnettozyma*, respectively).

In a study of mating success among 34 haplontic heterothallic species of *Metschnikowia*, Lee et al. (2018) determined that the ability to form mating pairs depended primarily on recognition of the α pheromone by cells of mating type **a**. A similar conclusion had been reached with mostly homothallic species of *Saccharomyces* and closely related genera (Rogers et al. 2015). In haplontic *Metschnikowia* species, completion of meiosis and ascosporulation only occur between mating pairs that exhibited little DNA sequence divergence (GSC). In this group of species, viable ascospores are formed in pairs, and asci arising from hybrid crosses give rise to large proportions of single-spored or empty asci. The single spores are generally sterile (Lachance and Bowles 2004; Marinoni and Lachance 2004). When recently diverged species are examined (Lee et al. 2020), a small amount of hybrid fertility may be observed, and the distinction becomes less clear. All other species examined in the *Metschnikowiaceae* share the same arrangement of mating genes and occur as haploid mating types or heterozygous diploids. Intriguingly, Muñoz et al. (2018) observed that isolates of *Candida auris* belong to distinct geographic clades, each of which features only one of the two known mating types. One has yet to report any attempts to mate the strains and observe ascospore formation in *C. auris*. Moving farther out into the larger CUG-ser-I clade, a few closely related species of *Kodamaea* with heterothallic haplontic life cycles do not exhibit any signs of hybridization (Lachance et al. 1999). Farther out yet, species of *Zygoascus* show a positive correlation between the formation of mature asci in crosses between the haploid strains and their DNA reassociation values, although many anomalies exist (Smith et al. 2005). In that case, interfertility served as an adjunct criterion for species circumscription.

One may rightly ask whether the BSC is in fact a genuine indicator that yeasts capable of fertile mating in the laboratory also belong to Mendelian populations in nature, which would contribute to maintaining their cohesion as evolutionary species. Five species of haplontic *Metschnikowia* have been examined for this very purpose. The endemic species *M. borealis*, *M. lochheadii*, *M. hawaiiensis*, and *M. hamakuensis*, which mate readily on agar media, exhibit independence among several genetic markers (Lachance et al. 2008, 2016; Wardlaw et al. 2009). In contrast, *M. ipomoeae*, characterized by a very low propensity for entering the sexual cycle, exhibits sharp linkage disequilibria and for all practical purposes, evolves clonally. A low but significant amount of disequilibrium was observed in *Kurtzmaniella cleridarum* (*Debaryomycetacae*, CUG-Ser-I clade), which can be accounted for by a less avid mating system and a larger separation between habitats, namely, beetles that live in cactus flowers in the Sonoran and Mojave deserts (Lachance et al. 2013).

### Lessons from *Cryptococcus*, *Tremellomycetes*, *Agaricomycotina*, *Basidiomycota*

In the *Basidiomycota*, sexual processes needed to fulfil the BSC are governed by mating types that are defined by two mating-type loci: the pheromone and pheromone receptor (*P/R*) loci that governs mating initiation, conjugation, and clamp cell fusion, and the *HD* locus that contains transcription factors, which regulates gene expression upon successful zygote formation to initiate downstream sexual developments, including hyphal growth, basidia formation and maturation, as well as sporulation (Ni et al. 2011; Heitman et al. 2013; Coelho et al. 2017; Sun et al. 2019a). Thus, successful sexual reproduction occurs typically between cells that possess divergent and compatible alleles at both the *P/R* and *HD* loci. When the two *MAT* loci are unlinked and segregate independently during meiosis, the species is defined as having a tetrapolar mating system; when the two *MAT* loci are linked, either physically or genetically, the species is determined to have a bipolar mating system. Transitions between tetrapolar and bipolar mating systems have occurred during the evolution of basidiomycetous species, and they involved loss of mating specificity of one of the two *MAT* loci, or chromosomal translocation leading to physical linkage of the two *MAT* loci (James et al. 2006; Wu et al. 2015; Sun et al. 2017, 2019a, b; Zhu et al. 2017).

While sequence similarity can be employed as a good indicator of whether two isolates with a certain level of sequence divergence belong to different species, it alone does not necessarily prove whether or not two isolates with diverging genomes represent fully established, reproductively isolated species. As in all other eukaryotes, speciation in *Basidiomycota* requires reproductive isolation and restricted gene flow between lineages, the extent of which is however not well studied. Reproductive barriers maintaining such speciation can be prezygotic, where sexual reproduction is prevented by geographical isolation or incompatible pheromone/pheromone receptor interaction; these barriers can also be postzygotic where the production of viable mating progeny is prevented due to genetic variation existing between the different species, leading to either failed meiosis or meiotic products that have compromised genetic compositions that render them inviable. For example, in the human pathogenic *Cryptococcus* species complex, at least eight species are currently recognized using PSC/GCPSR that belong to three sub-complexes: *Cr. neoformans*, *Cr. deneoformans*, and *Cr. gattii* (Hagen et al. 2015; Farrer et al. 2019; Bahn et al. 2020). Genome analyses have shown that significant genetic variation has accumulated between these different species and that each species forms a well-supported clade with limited inter-specific gene flow, consistent with the existence of reproductive isolation between these species (Sun and Xu 2009; Desjardins et al. 2017; Rhodes et al. 2017). Thus, this is a nice example where the PSC-GCPSR/BSC and GSC largely coincide, while also phenotypic differences occur between these species, thus there is also support using the PhenSC. Interestingly, mating can still occur between certain *Cryptococcus* species and produce sexual structures that are morphologically indistinguishable from those arising from canonical intra-species mating, with fused clamp cells and abundant meiotic spores, suggesting that complete prezygotic reproductive isolation has yet to be established between these species. However, further analyses of the meiotic progeny revealed that they have very low germination rates and most of the viable spores are either aneuploid or diploid, suggesting that meiosis during inter-species mating has been significantly compromised, which is consistent with the fact that both high-level DNA sequence divergence and significant chromosomal rearrangements (e.g., inversions and translocations) are present between different *Cryptococcus* species. These divergent genomic properties collectively impede proper chromosomal pairing and recombination during meiosis and promote chromosomal mis-segregation (Sun and Xu 2007).

Gene flow can only be detected in population genetics/genomics data, which presents a problem when only a limited number of isolates of a species are available. In a recent study of four isolates that represent three non-pathogenic *Cryptococcus* species, *Cr. amylolentus*, *Cr. floricola*, and *Cr. wingfieldii*, which are closely related to the pathogenic *Cryptococcus* species complexes, the authors first obtained chromosome-level genome assemblies using PacBio and Nanopore long-read sequencing and de novo genome assembly. Subsequent genome comparisons revealed that while these non-pathogenic species share 93.5–94.4% sequence similarity, extensive chromosomal rearrangements, including inversions and translocations, are present, suggesting that they could represent distinct species. Additionally, these species have *MAT* loci configurations consistent with a tetrapolar mating system and possess divergent *MAT* alleles, indicating they should be mating compatible with each other, as confirmed by previous studies of the two *Cr. amylolentus* isolates (Findley et al. 2012). However, successful mating was only observed between *Cr. amylolentus* and *Cr. floricola*, and their meiotic progeny were either inviable or sterile, and showed reduced recombination during meiosis, suggesting postzygotic barriers had been established between the two species. Furthermore, physiological characterization of these three species revealed they have distinguishing phenotypic profiles that might reflect the differences in their natural habitats. Thus, multiple lines of evidence collectively support that *Cr. amylolentus*, *Cr. floricola*, and *Cr. wingfieldii* represent distinct species (Passer et al. 2019). A good correlation between BSC, PhenSC and PSC was observed in that study.

It should be noted that in some cases, isolates belonging to the same species with compatible mating types based on genome analyses fail to undergo sexual reproduction. It is possible that the laboratory conditions are not ideal to induce mating, or that the isolates might be sterile due to changes in the genes required for mating. Because sexual reproduction is not obligate in most basidiomycetous fungi, sterility is disadvantageous in the short term only if those genetic changes have pleiotropic effects that reduce the hosts’ vegetative fitness, although the inability to undergo sexual reproduction is almost always detrimental during long-term evolution.

As mentioned earlier, both DNA sequence divergence and chromosomal rearrangements contribute to compromised meiosis and the establishment of postzygotic reproductive isolation between species. Because these two types of genetic variation almost always exist simultaneously when different species are compared, it has been difficult to dissect the effect of one in the absence of the other. In a recent study of *Cr. neoformans*, the authors induced double-strand breaks (DSBs) at the centromeres using CRISPR, and the subsequent repair of these DSBs by the cell resulted in a strain in which a large number of chromosomal arms were reshuffled, while all of the genetic information in the genome had been retained (Yadav et al. 2020). Genetic crosses between the original *Cr. neoformans* strain and the strain with a reshuffled genome revealed that while mating could still occur, the crosses produced almost no spores, suggesting chromosomal structural variation alone could be sufficient to establish a postzygotic reproductive barrier, although both sequence divergence and chromosomal rearrangements likely impose reproductive barriers collectively between closely related species in nature (Yadav et al. 2020).

There are also factors that enable species to overcome postzygotic reproductive barriers. For example, it has been shown in yeast species that the mismatch repair system is involved in repressing homeologous recombination between divergent sequences, and thus preventing chromosomal pairing and crossing over during hybridization, leading to compromised meiosis and chromosomal mis-segregation. Deletion of key genes in the mismatch repair pathway, such as *MSH2*, could significantly increase the spore viability during inter-species hybridization (Chambers et al. 1996; Hunter et al. 1996). It has been shown recently that in *Cryptococcus* deletion of *MSH2* also leads to increased spore viability during inter-species hybridization, even without significantly elevated recombination frequency during meiosis (Priest et al., 2021). Additionally, while sequence divergence and chromosomal rearrangements are detrimental during meiosis when the two isolates involved in the hybridization are haploid, diploidization of both isolates could significantly alleviate such deleterious effects because in this case all of the chromosomes exist in identical pairs and thus, are able to form homologous chromosomal pairs during meiosis I (Greig et al. 2002). Whether such factors also exist in basidiomycetous species has yet to be determined.

### Lessons from *Ustilaginomycotina*, *Basidiomycota* and *Microbotryales*, *Pucciniomycotina*, *Basidiomycota*

Classically, the ‘smuts’ comprise a group of fungi that are host-specific plant parasites that infect a large range of plant taxa. They exhibit a typical life cycle, whereby a basidium germinates from a diploid teliospore and produces haploid basidiospores, which can reproduce asexually by budding (Begerow et al. 2014). These asexually produced yeasts are also called sporidia. To initiate the plant parasitic phase, two basidiospores/sporidia with compatible mating types need to fuse on a suitable host and produce a dikaryotic infection hypha. After successful penetration of the plant epidermis through an appressorium the fungus proliferates within its host as a mycelium. At a given developmental stage of the host, the fungus starts forming spore-producing structures (i.e., sorus; pl. sori), which at maturity erupt from the plant tissue. In the majority of cases, sori are plant tissue specific for a given smut fungal species (Schäfer et al. 2010; Begerow et al. 2014). The combination of host plant and place of soral development together with other morphological characteristics (e.g., spore mass colour) are used for species identification in the field. The systematics and understanding of evolution of smut fungi have been mainly based on these characteristics, as well as on the additional morphological traits spore size and spore ornamentation (Vánky 2012), thus implying the applications of the phenotypic and ecological species concepts.

However, recent years have seen a revolution in the understanding of smut fungus evolution and systematics that has been driven by application of molecular data. The smut fungi themselves have been recognized as a polyphyletic group and fungi with the smut life history have evolved in both *Ustilaginomycotina* and the *Microbotryales* (*Pucciniomycotina*). In addition, it is now known that many species of *Ustilaginomycotina* appear to be yeasts without a known parasitic stage (e.g., *Malasseziomycetes* and *Moniliellomycetes*) (Begerow et al. 2000; Wang et al. 2014, 2015a) where, by default, the BSC cannot be applied. Because of the different life history strategies employed within *Ustilaginomycotina*, no single species concept has been applied within this group. While the PhenSC is still relevant for species delimitation and identification, it does not provide a conceptual idea of what a species actually represents, and which mechanisms maintain its cohesion. Species recognition of ‘smuts’ is mainly based on the PSC using molecular data, but also incorporating host taxa as an autapomorphy, thus leading to an integrative species concept. As with the phenotypic concept, a strict application of the PSC does not readily incorporate biological data. Therefore, investigating the BSC is the basis for studies focusing on mechanisms involved in the maintenance of species integrity in smut fungi. Several research lines related to the BSC thereby have received most attention: a. Function and evolution of smut fungal mating systems; b. Host adaptation as a mechanism important in creating and maintaining species; and c. The role of asexual lineages in *Ustilaginomycotina*. Aspects of hybridization of smut fungi are discussed in the appropriate text below.

#### Function and evolution of smut fungal mating systems

Like most members of the *Basidiomycota*, smut fungi use a mating system consisting of two relevant factors. The *MAT* locus represents a pheromone/pheromone receptor (*P/R* locus) system, where one strain expresses the pheromone that interacts with the pheromone receptor of a second strain. The second locus consists of a homeodomain transcription factor formed by a heterodimer (*HD* locus), where both subunits are expressed by two different strains. While the *P/R* locus is essential for finding the mating partner of a compatible mating type, the HD locus confers the maintenance of the dikaryon and the proper development of hyphae (Bölker et al. 1992; Gillesen et al. 1992; Lee et al. 2010; Coelho et al. 2017). Both factors are involved in the development of the parasitic phase (Bölker et al. 1995; Kämper et al. 2020). All smut fungi are parasitic in the dikaryotic phase only and therefore mating genes were identified as virulence factors (Brachmann et al. 2001; Kahmann and Schirawski 2007). Current research indicates that both factors are part of all major lineages and are probably similar in function (Kijpornyongpan and Aime 2020). Although central to the maintenance of species, the mating system has undergone several modifications during the evolution of *Basidiomycota*. For instance, the number of alleles and the linkage of these factors varies significantly (Kellner et al. 2011). Many studies have focused on the presence and absence of bipolar and tetrapolar mating systems, which result from a recombination block between the two mating loci and transitions between the two mating systems seem quite common (Lee et al. 2010). Comparative genomics studies of smut fungi revealed a broad spectrum of evolutionary variations, including chromosome rearrangements and recombination suppression (Branco et al. 2017; Sun et al. 2017; Kijpornyongpan and Aime 2020; Yadav et al. 2020), and multi-allelic mating loci having tri-allelic pheromone loci (Kellner et al. 2011). Only a few species and lineages have been studied in this respect so far, but the implications for the BSC are already complex. Like in other *Basidiomycota* two haploid genotypes with compatible mating types fuse to form a pathogenic dikaryon. On the molecular level, however, smut fungi have evolved a plethora of mating systems to enable successful mating between individuals, all of which maintain the main function, i.e. the creation of new genotypes.

#### Host adaptation as a mechanism important in creating and maintaining species

Reproductive isolation of well-adapted gene pools protects them from the invasion of non-adapted genetic material and is central to the BSC (Mayr 1969, as cited in Mayr 1996). Detecting and understanding mechanisms that generate and maintain this isolation are therefore a central point in research on biological species. Most *Ustilaginomycotina* and *Microbotryales* are parasitic and the host-parasite interaction has most likely resulted in a high host specificity (Begerow et al. 2014; Hartmann et al. 2019), and as such this fits the Ecological Species Concept. While co-evolution has been used to explain host specificity of smuts (Begerow et al. 2004), host jumps are also quite common, not only on closely related hosts, but also between different plant families (Kemler et al. 2020). In some cases, two or more species have adapted to a single host species and can co-occur in the same host population without a disintegration of parasite species delimitations (Begerow et al. 2002; Abbate et al. 2018). Studies focusing on virulence and pathogenicity factors are trying to identify factors conveying host specificity (e.g., Ökmen et al. 2018). However, as genes responsible for host adaptation are not necessarily involved in sexual reproduction, the way in which host adaptation plays a role in reproductive isolation and speciation of smut fungal populations on different hosts is still an active field of research. Phylogenetic studies support the hypothesis that host adaptation and speciation are linked, as the vast majority of smut fungi on different host species form evolutionary independent lineages (e.g., Begerow et al. 2002; Lutz et al. 2005; Stoll et al. 2005; Piątek et al. 2013; Denchev et al. 2019). On the other hand, work on smut fungal populations shows ongoing gene flow between closely related parasite species (Petit et al. 2017) and experimental work clearly indicates that even distantly related species are able to hybridize (e.g. Büker et al. 2020; Kellner et al. 2011). High selfing rates by fusion of meiotic products from the same teliospore, as is for instance common in *Microbotryum* anther smuts (Giraud et al. 2005), could restrict gene flow of sympatric populations on different host species enough for reproductive barriers between species to evolve on different hosts (Giraud et al. 2008). Thus, while host specificity is still important to identify species in the field, its role in generating and maintaining biological species remains unclear.

#### The role of asexual lineages in the smut fungi

One obvious pitfall of the BSC is that it offers no assistant in delineating asexual species. Agamotaxon has been suggested by cladists as a term for uniparental lineages to indicate different mechanisms that maintain well-adapted genomes (Meier and Willmann 2000), but this convention is not widely used by mycologists. The *Ustilaginomycotina* contain numerous asexual yeasts that reproduce by budding, such as *Acaromyces* spp., *Meira* spp., *Tilletiopsis* spp. in the Exobasidiomycetes, *Pseudozyma* spp. and the *Violaceomycetales* in the *Ustilaginomycetes*, *Malassezia* in the *Malasseziomycetes*, and *Monilliella* in the *Monillielomycetes* (Boekhout et al. 2003; Rush and Aime 2013; Begerow et al. 2014; Wang et al. 2014, 2015a; Albu et al. 2015). For most of these lineages it is unknown whether the sexual stage has been found, whether these lineages have lost the sexual stage, or if sexuality takes on a different form compared to other *Ustilaginomycotina*. As with other crucial questions concerning basic fungal biology, DNA sequencing and especially whole genome sequencing (WGS) gave important insights into potentially asexual smut fungal lineages. Molecular phylogenetic analyses have shown that several of the asexual yeast taxa found ubiquitously in the environment represented the asexual stages of known smut fungi. The commonly isolated *Pseudozyma prolifica* for instance has been shown to be the anamorphic state of the well-known smut fungus *Mycosarcoma maydis* (formerly known as *Ustilago maydis).* Additionally, other yeast lineages are con-generic with well-known smut fungal genera in *Ustilaginomycotina* and *Microbotryales* (Begerow et al. 2000, 2014, 2017; Wang et al. 2015a, b), but whether they represent plant parasitic smut fungi is not known. WGS gives good indications that several of these ‘asexual’ species, potentially have the possibility for sexual reproduction. The genes controlling sexual reproduction in *Ustilaginomycotina* and *Microbotryales* have been identified (see mating type evolution, above) and analyses of whole genomes showed that potentially functional mating types are present in several asexual taxa (Coelho et al. 2017), including *Malassezia* species (Xu et al. 2007; Wu et al. 2015). Nevertheless, in some lineages (e.g., *Violaceomyces palustris*) essential components of the *P/R* locus, such as pheromone receptors and their pheromone precursors, are lacking (Kijpornyongpan and Aime 2020), indicating that not all smut fungal taxa undergo sexual reproduction.

## Hybridization and species concepts in yeasts

Fungi are broadly used as experimental systems to detect and study hybridization. Both the species concept itself and species delimitation are challenged by the existence of hybrids. The term hybridization was coined in biology to define individuals resulting from the cross of genetically distinct organisms, without necessarily referring to organisms of different species, e.g., Gregor Mendel used the word to refer to the progeny resulting from crossing different breeds of a single pea species, *Pisum sativum* (Mendel 1866). Nowadays, the definition of hybrids is commonly related to that of species, distinguishing between intra- (within) or inter- (across) species hybrids (Abbott et al. 2013). Here, we shall discuss several outstanding issues that complicate the use of a species concept in hybrid species/lineages.

Inter-species hybrids are common among yeasts. They are not only present as recently formed organisms in industrial, clinical, or environmental niches (Morales and Dujon 2012; Mixão and Gabaldón 2018)⁠, but also can be at the root of established lineages (Gabaldón 2020a)⁠. For instance, species such as the important opportunistic pathogen *Candida albicans*, or the so-called post whole genome duplication clade of *Saccharomycotina* species have likely originated from hybridization events (Wolfe and Shields 1997; Dietrich et al. 2004; Marcet-Houben and Gabaldón 2015; Mixão and Gabaldón 2020).

Due to technical improvements in genome analysis and sequencing technologies, bio-informatics, as well as awareness, more yeast hybrids are recognized to occur in various yeast lineages, e.g. in the *Cryptococcus neoformans/Cr. gattii* complex (Boekhout et al. 2001; Bovers et al. 2006, 2008; Samarasinghe and Xu 2018) and *Trichosporonales* (*Basidiomycota, Agaricomycotina, Tremellomycetes*) (Sriswasdi et al. 2019), *Malassezia* (*Basidiomycota, Ustilaginomycotina, Malasseziomycetes*) (Wu et al. 2015), *Candida* (*Ascomycota, Saccharomycotina*) (Mixão and Gabaldón 2018, 2020; Mixão et al. 2019; Pryszcz et al. 2014), *Pichia* (*Ascomycota, Saccharomycotina*) (Leh-Louis et al. 2012), and *Schizosaccharomyces* (*Ascomycota, Taphrinomycotina*) (Zanders et al. 2014). These hybrids may be important in applied areas as they may have beneficial properties e.g., in fermentations, but similarly this may also result in novel virulent lineages (Gabaldón 2020a; Morales and Dujon 2012). Hybridization may be followed by extensive post-zygotic genome modification, such as loss of heterozygosity (LOH), structural mutations and point mutations, and copy number changes resulting in fitness differences (Piotrowski et al. 2012, Smukowski Heil et al. 2017). See also below for more on this topic.

### Hybrids in the *Saccharomycotina*

Non-vertical evolutionary processes, such as introgression and hybridization, may complicate the proper recognition of species (Gabaldón 2020a; see below). This, of course, may cause inconvenience for the investigator, but eventually will result in a better understanding of the nature of species and how to utilize such information. An illustrative example is the brewer’s yeast *Saccharomyces pastorianus*, including its synonym *S. carlsbergensis* that is widely used for the production of lager beers. Initially believed to be a unique species, *S. pastorianus* was later found to be the result of a hybridization event between *S. cerevisiae* and *S. eubayanus* (Libkind et al. 2011). Following the discovery of the hybrid nature of this species subsequent research focused on how to utilize this knowledge in the beer making process (Langdon et al. 2019). Another example is *Saccharomyces bayanus*, a hybrid involving contributions from three distinct species as parents: *S. cerevisiae, S. eubayanus*, and *S. uvarum* (Libkind et al. 2011; Pérez-Través et al. 2014). The proper recognition of this hybrid resulted in the circumscription of *S. uvarum* as a genetically well-defined species (Nguyen and Gaillardin 2005; Nguyen and Boekhout 2017).

Contrary to the situation in plants or animals, where hybrids are traditionally recognized by the presence of intermediate phenotypic traits between well-defined parental species, hybrids in yeasts are usually first recognized upon analysis of several genomic markers or complete genomic sequences (e.g., Boekhout et al. 2001; Nguyen and Gaillardin 2005; Nguyen and Boekhout 2017; Gabaldón 2020b, Naranjo-Ortiz and Gabaldón 2020). This means that the parental species that originated the hybrid are not necessarily known. If the parental species are described and sequenced, the description and naming of the hybrid is straightforward. However, in many cases only one of the parental species, or none is known, and their existence can only be inferred from the divergence of the two hybrid sub-genomes. To illustrate this issue, we will refer to two hybrid lineages within the *Candida parapsilosis* species complex (Pryszcz et al. 2014, 2015) as well as three hybrids of *Trichosporonales*, *Basidiomycota* (see below Takashima et al. 2018, Sriswadi et al. 2016, 2019). Although strains within the *C. parapsilosis* clade were initially described as a single species, *Candida parapsilosis*, this clade was re-organized into three distinct species, *C. parapsilosis* (sensu stricto), *C. orthopsilosis*, and *C. metapsilosis* (Tavanti et al. 2005). Further sequencing of their genomes revealed that both *C. orthopsilosis* (Pryszcz et al. 2014; Schröder et al. 2016) and *C. metapsilosis* (Pryszcz et al. 2015) comprised, respectively, a majority or exclusively hybrid strains. This raises the issue of whether the hybrid strains of *C. orthopsilosis*, which are the result of the crossing of the lineage represented by the type strains and an unknown parental, should be considered a different species and given a different name. The situation of *C. metapsilosis* is somewhat different as the type strain, which served as the basis for describing the species, is a hybrid itself, and none of the sequenced strains seem to represent a pure, homozygous lineage. Hence two different parental lineages can be inferred, but these are (thus far) unknown, a situation that precludes their formal description, at least using standard approaches. Another issue relates to the parallel emergence of hybrids. Species that can form successful hybrids by crossing can form the same hybrids multiple times. This seems to be the case in various hybrids such as *C. orthopsilosis*, where at least four independent events of hybrid formation can be inferred (Schröder et al. 2016). Shall one consider independently formed hybrids between the same parental lineages as the same species or as different ones? If the former assumption is made, this will mean that the same species can originate several times and in different locations independently. This concept of convergent species formation is at odds with our standard understanding of the origin of species. Finally, the definition of a hybrid lineage as a new species is clearly rooted in the very definition of species, as hybrids between different species may be considered as candidates for new species, whether intra-species hybrids may be better accommodated in the concept of variety.

Hybrids have been extensively found in the genus *Saccharomyces*, including *S. pastorianus/carlsbergensis* mentioned above, but also *S. cerevisiae* x *S. kudriavzevii, S. uvarum* × *S. eubayanus* and others indicating that interspecies *Saccharomyces* hybrids can form readily. Although there is some evidence of prezygotic reproductive isolation (Greig 2009; Ono et al. 2020) most of the reproductive isolation between species is post-zygotic that is the basis of the BSC (see above). Population genomics surveys reveal that introgression between species occurs regularly (Dujon and Louis 2017) with one of the first described being between *S. cerevisiae* and *S. paradoxus* (Liti et al. 2006) where the European population of *S. paradoxus* contains a segment of *S. cerevisiae* sequence as a homologous replacement of the *S. paradoxus* sequences found in other populations. This indicates that reproductive isolation is not complete and that some gene flow between species can occur, generally thought to be due to a rare viable spore being successively backcrossed to one of the parents. The spore viability of hybrids between *Saccharomyces* species is generally around 0.5% which is the expected frequency of obtaining a viable set of chromosomes when 16 pairs are randomly segregating. The chromosomes in these viable spore progenies have few if any crossovers and tend to be aneuploid. In *S. cerevisiae* × *S. paradoxus* hybrids, where there are no GCRs, no nuclear Bateson–Dobshansky–Muller incompatibilities have been found (Greig et al. 2002; Greig 2007; Kao et al. 2010). Instead, fertility is increased when the mismatch repair system is defective (Chambers et al. 1996; Hunter et al. 1996; Greig et al. 2003), which results in an increase in meiotic crossovers and a decrease in mis-segregation of chromosomes. As the mismatch repair system has crucial functions throughout the lifecycle of yeast, complete fertility restoration was not possible. Recently, using a reduction of mismatch repair specifically in meiosis along with meiotic loss of the *SGS1* helicase which unwinds most meiotic recombination intermediates as well as has crucial mitotic functions, Greig and colleagues were able to restore most of the fertility of hybrid spores (from 0.5% to over 30%) successfully breaking down the post-zygotic species barrier (Ozan Bozdag et al. 2021). Given that the major reproductive isolation mechanism leading to species in *Saccharomyces* appears to be the lack of recombination and the resulting missegregation of homeologous chromosomes, how do we explain introgressions that are found between species throughout the genus? In a hybrid between *S. cerevisiae* and *S. uvarum* under selective conditions, a recurrent rearrangement was found that included the introgression of a segment of the *S. uvarum* genome into the *S. cerevisiae* genome (Dunn et al. 2013). If a viable spore from this modified hybrid contained mostly *S. cerevisiae* chromosomes or the *S. uvarum* genome was lost as can happen in unstable hybrids, a rapid introgression into the *S. cerevisiae* population can occur without numerous backcrosses. Very recently a number of isolates from the Alpechin population of *S. cerevisiae* were analysed by whole genome sequencing (Peter et al. 2018) and these contain a large number of introgressions from *S. paradoxus* (up to 8% of the genome in some isolates). In this population an *S. cerevisiae* × *S. paradoxus* hybrid was found that upon whole genome sequence analysis and proper haplotyping was found to be the living ancestor of the strains with *S. paradoxus* introgressions (D’Angiolo et al. 2020). This hybrid has suffered massive genome instability with mitotic recombination between the homeologues and many regions of loss of heterozygosity such that every chromosome pair now has homologous regions interspersed between the homoelogous regions. This living ancestor has one *S. cerevisiae* genome with numerous small introgressions from the *S. paradoxus* genome along with one *S. paradoxus* genome with a few introgressions from the *S. cerevisiae* genome. The introgressions found in the extant Alpechin *S. cerevisiae* population are found in the living ancestor’s *S. cerevisiae* genome. The now homologous segments are sufficient to allow meiotic recombination between the homeologues and proper segregation resulting in some viable spores. This fortuitous finding of the progenitor hybrid in an intermediate state with the introgressions indicates how extant populations across the *Saccharomyces* clade may have arisen and reconciles the paradox of introgressions in species whose reproductive isolation barrier is lack of homologous recombination.

### Hybrids in the *Microbotryales*, *Pucciniomycotina*, *Ustilaginomycotina* and the *Tremellomycetes*, *Agaricomycotina*

Population studies of *Microbotryales* smut fungi revealed that several members can hybridize and gene flow between species occurs (Devier et al. 2010; Gibson et al. 2014; Petit et al. 2017; Hartmann et al. 2020). A study of these pathogens even indicated the relevance of hybrids in the development of new virulence patterns (Büker et al. 2013). Kellner et al. (2011) showed that non-specificity in the pheromone/pheromone receptor system of *Ustilaginales* might provide the molecular basis for such hybrids. However, pre- and postzygotic reproduction barriers in agreement with the BSC are the norm in these fungi, as even artificial mating experiments in the laboratory resulted in few viable hybrids. Still, the hybrids that survived showed rare recombination events (Büker et al. 2020), which could be important for accessing new hosts and the evolution of new smut fungal species.

Hybrids between different pathogenic *Cryptococcus* species, in particular between *Cr. neoformans* and *Cr. deneoformans*, have been isolated from the clinic and the environment, suggesting that inter-species hybridization may still occur in nature (Xu et al. 2000, 2002; Boekhout et al. 2001; Lengeler et al. 2001; Bovers et al. 2006, 2008; Lin et al. 2007; Litvintseva et al. 2007). *Cryptococcus* hybrids can still reproduce asexually as yeast and in some cases have higher fitness compared to haploid *Cryptococcus* species due to their increased ploidy level (aneuploid/diploid) and hybrid vigor arising from heterozygosity (Lin et al. 2007, 2008).

Recently, whole genome analysis has shown that three species of *Trichosporonales*, *Trichosporon coremiiforme*, *T. ovoides* and *Cutaneotrichosporon mucoides* are hybrids (Takashima et al. 2018, 2019). The genomes of *Cu. mucoides*, *T. coremiiforme* and *T. ovoides* were estimated to be 40.8, 42.35 and 40.3 Mb in size, respectively, which is significantly larger than those of putative haploid genomes of *Trichosporonales*, which range from 17.2 to 36.6 Mb and the number of genes of the three hybrids ranged from 12,979 to 14,292 compared to 5,647–9,805 in the putative haploid species (Takashima et al. 2018, 2019, Aliyu et al. 2020). A bioinformatics analysis showed that the two subgenomes of *Cu. mucoides* were most closely related to that of *Cu. dermatis*, with subgenome A being closest with 85% similarity. The subgenomes of *T. ovoides* were most closely related to that of *T. inkin* with subgenome A being the closest. The two subgenomes of *T. coremiiforme* yielded a surprise as subgenome A was found to be closely related to the genome of *T. asahii*, whereas subgenome B was closer to the genome of *T. faecale*. It was estimated that the *T. ovoides* and *T. coremiiforme* hybrids originated 14–22 Mya, which is much older than the recently formed hybrids of *Saccharomyces* species. The authors made a distinction between recently formed hybrids such as those occurring in *Saccharomyces*, the so-called Class I hybrids, and those that originated in a distant evolutionary past, the so-called Class II hybrids. The authors considered that the latter could be interpreted as species (see also below), and in several cases they were already described as separate species—see the species of *Trichosporonales* and those of the *C. parapsilosis* complex. The close phylogenetic relatedness between haploid and hybrid species in, e.g., the *Trichosporonales* hybrids, may be the reason for the difficulty of identifying them by MALDI-TOF MS that targets the proteome (Kolecka et al. 2013).

An in-depth sequence comparison and a transcriptome analysis of two of these *Trichosporonales* hybrids and their putative parental ancestors yielded some insights in the post-hybridization processes of the three hybrids (Sriswasdi et al. 2016, 2019). The genome comparisons revealed that redundant genes underwent a deceleration, and not an acceleration of their evolutionary rates. It was suggested that gene conversion might have contributed to the genome stability of the initial unbalanced hybrid genomes by restricting the functional differences between homologous gene sites from both subgenomes present in the hybrid. Moreover, large-scale gene loss, in particular from the transcriptional and translational machineries, formed a global compensatory mechanism against increased gene dosages resulting from the hybridization events (Sriswasdi et al. 2019). In the other study it was observed that the genome and transcriptome evolved separately after the hybrids were formed. Moreover, the various hybrids showed differences in the involvement of both subgenomes in the massive gene loss, where either only one or both subgenomes were involved (Sriswasdi et al. 2019). It was concluded that the stabilization of the genomes and the transcriptomes are two different evolutionary processes in young allopolyploids, and that closely related hybrids may follow different evolutionary trajectories (Sriswasdi et al. 2019).

Gabaldón (2020b) has proposed three Hybrid Genetic Zones (HGZ) defined by the interaction between gene flow and genetic divergence to differentiate between various hybrids. Sexually reproducing species show high gene flow and limited genetic divergence and crosses yield highly viable progeny. HGZ 1 hybrids show a relatively high gene flow with relatively low genetic divergence. These hybrids occur relatively often, and their progeny has moderate to low viability. Recently formed hybrids among *Saccharomyces* species and the *Cryptococcus neoformans* x *Cr. deneoformans* hybrids, which also can be generated easily in the laboratory, may belong here. HGZ 2 hybrids are characterized by limited gene flow and limited genetic divergence. It is probable that *Cu. mucoides* belongs in this category as its genes were still 85% identical with those of its ancestor *Cu. dermatis* (Takashima et al. 2018). Several of the hybrids described within *Candida* species with genetic divergences ranging from 3 to 5%, such as *C. orthopsilosis* (Pryszcz et al. 2014), *C. metapsilosis* (Pryszcz et al. 2015) or *C. inconspicua* (Mixao et al. 2019). Finally, *C. albicans* may represent a highly evolved HGZ 2 hybrid, in which continued LOH has extensively reduced the amount of heterozygous regions (Mixão and Gabaldón 2020). Finally, HGZ 3 hybrids are characterized by extensive genetic divergence and moderate gene flow. These hybrids might occur more rarely, and may undergone rapid ploidy changes and losses of one of the subgenomes to minimize genomic incompatibilities. *Millerozyma (Pichia) farinosa,* resulting from the hybridization of two parental species diverging > 10% at the nucleotide level may represent a hybrid of this class (Leh-Louis et al. 2012). Note that hybrids categorized in each of the HGZ classes from Gabaldón may correspond to one of the two temporal classes proposed by Takashima and collaborators, as both categorizations focus on properties that are orthologonal to each other (divergence of the hybridizing species in the former, or how long ago the hybrids were formed in the latter).

Based on these observations, one can state that the genetic background of various yeast hybrids may differ widely. Hybrids belonging to class II/HGZ2/3 may represent (small) speciation networks whose ancestral species may or may not be known. Moreover, the demonstration of a hybrid origin of the whole genome duplication lineage of *Saccharomyces* (Marcet-Houben and Gabaldón (2015), as well as the hybrid origin of *C. albicans* (Mixão and Gabaldón 2020) and *C. stellatoidea* (Mixão et al. 2021) clearly demonstrate that hybridization may also drive speciation.

## The role of DNA sequences in the practical recognition of yeast species

From the above it is clear that the advent of DNA sequencing has had an unprecedented impact on the recognition of yeast species. Nucleotide sequence databases such as NCBI Taxonomy (https://www.ncbi.nlm.nih.gov/Taxonomy/taxonomyhome.html/), UNITE (https://unite.ut.ee/), and theyeastst.org (https://theyeasts.org/) can be used to report validly described species, as well as unassigned strains and sequences from clone libraries and metabarcoding. It is important to realize that these databases are prone to contamination with inaccuracies in the sequences, the taxonomy, or both. We are greatly indebted to C.P. Kurtzman for his vision of giving the highest priority to populating a complete database of a single orthologous genomic region using type and reference strains, i.e., the D1/D2 domains of the Large Subunit (LSU) rRNA gene (Kurtzman and Robnett 1998). The sequence database may also serve as a forum that can bring together various authors to share their respective knowledge of a novel species. Databases can be exploited for automated and semi-automated data mining on species distributions across geography and habitats, and the evaluation of sequence polymorphisms. The counterpart is that sequences are so easily accessible that the effort needed to document morphology and growth responses seems enormous in comparison. Yet, if the description is to achieve its full purpose, *it must describe*. The cycler cannot replace the microscope or the fermentation tube, and a sequence is not a living organism.

Sequencing technologies have stimulated the design of sophisticated algorithms and associated software implementations. Because the various tasks of systematics are both similar and distinct, the systematist must seek to use appropriate methods. At the very start of the process, online searches for a ranked list of the best matching sequences are subject to the vagaries of artefacts specific to the laboratories that generated both the query and the target sequences (Stavrou et al. 2018; Pentinsaari et al. 2020). The default ranking of a BLAST search (Altschul et al. 1990) is a function of both identity and query coverage, such that the rank order may not truly reflect relatedness. Depending on the length of the compared sequences, different BLAST algorithms (megablast, discontinuous megablast and blastn) can yield different orders of best matches (discussed in Lücking et al. 2020). Once a suitable set of published sequences is selected for further analysis, a competent alignment must be generated, the details of which are beyond the scope of this article (but see Lücking et al. 2020, 2021). The construction of a tree then follows with the dual purpose of (1) identifying the phylogenetic position of the novel species in the hierarchy of classification and (2) adding evidence that a new species has indeed been discovered. The first task must be regarded as preliminary, as a robust phylogeny simply cannot be obtained from insufficient data (in terms of both sequence data and taxon sampling), no matter what tree inference algorithm is used (Aime et al. 2021). The second objective must consider what species concept is to be applied. If the BSC is to be given precedence, then the kind of phylogenetic tree is irrelevant. But if, as is usually the case with yeasts, the argument is founded primarily on Kurtzman’s GSC, the selected tree must retain distance information in the form of terminal branch lengths (the maximum likelihood method often truncates terminal branches).

Since the introduction of molecular phylogenetic datasets and methods it has become clear that many previously recognized phenotypic species represent species complexes/cryptic species sharing similar morphological and growth characters. Examples are the *Cr*. *neoformans*/*Cr*. *gattii* species complex (Hagen et al. 2015, 2017; Kwon-Chung et al. 2017), *Papiliotrema flavescens*/*P. terrestris* species complex (Yurkov et al. 2015), *C. albicans*/*C*. *dubliniensis* (Sullivan et al. 1995), and lipid dependent *Malassezia* species (e.g., *M*. *arunalokei*, *M*. *restricta*, *M*. *globosa*) (Guého et al. 1996; Honnavar et al. 2016). In the case of such cryptic species, the BSC and/or the PSC/GCPSR are helpful to understand species delineation. (Giraud et al. 2008; Taylor et al. 2000). GCPSR can recognize cryptic species that lack distinguishing phenotypic features and that do not mate due to prezygotic (i.e., pre-mating) isolation, and in which inferences on the BSC can only be made indirectly, e.g., by showing recombination using molecular tests (Giraud et al. 2008; Hagen et al. 2015; Taylor et al. 2000).

### The value of barcodes for species identification

**Fig. 2:**
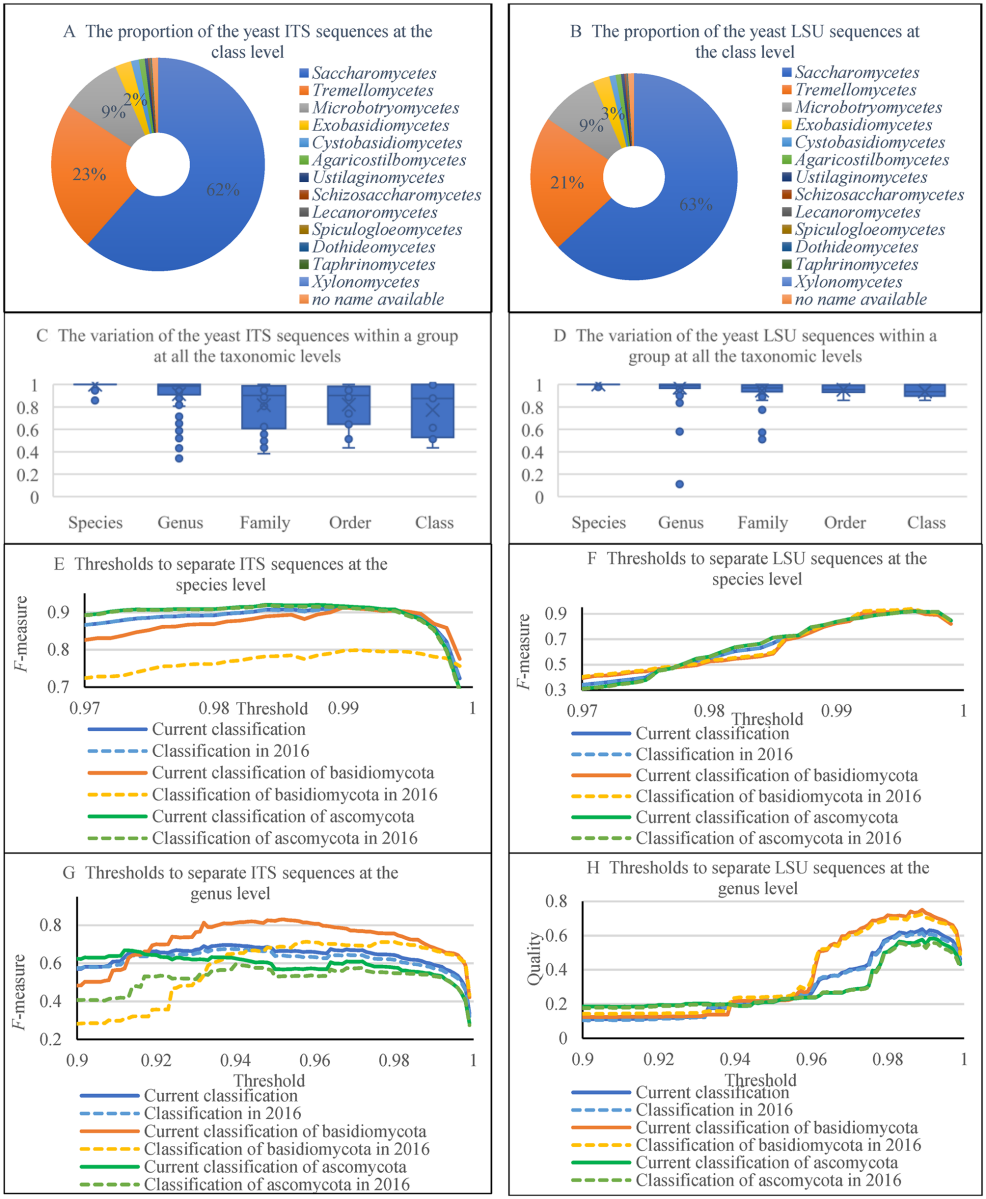
Analysis of ITS1 + 2 and D1/D2 LSU ribosomal RNA gene sequences of yeasts. **A**, **B** Proportion of yeast ITS and LSU sequences at the class level. **C**, **D** Variation of the median ITS and LSU sequence similarity scores of yeasts at various taxonomic levels. **E**–**H** Optimal thresholds and associated highest *F*-measures predicted at the species and genus levels from a previous analysis (Vu et al. 2016) and a current dataset updated based on recent taxonomic revisions (date of analysis December 2020). The sequences were compared with each other using BLAST (Altschul et al. 1997). For each of the resulting local alignments of two sequences, a BLAST similarity score was calculated as the percentage of matches *s* if the alignment length *l* was greater than 300 bp (the minimum length of ITS sequences, Vu et al. 2019). Otherwise, the score was recomputed as $sl/300$. The names of the taxa associated with the sequences were downloaded from MycoBank (Robert et al. 2013)

**Fig. 3:**
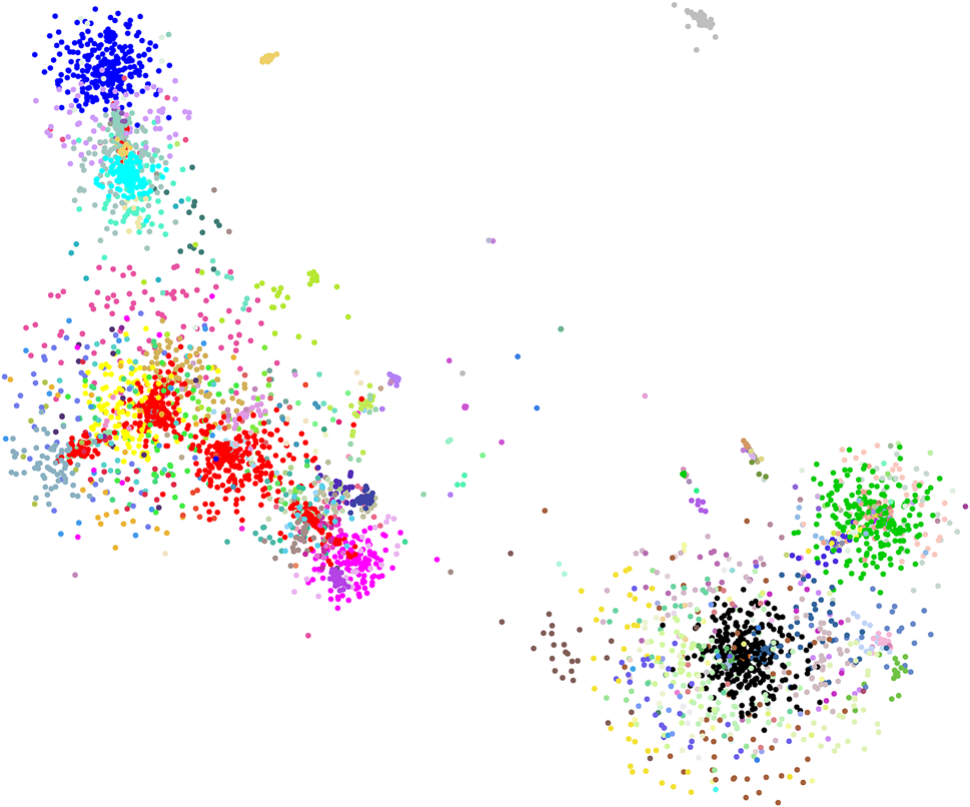
Taxonomic clustering of 4,356 fungal ITS barcodes of the current taxonomic classification. The sequences are coloured based on the genus name. *Candida* (with 593 sequences) is indicated in red, followed by *Cryptococcus* (329) in black, *Saccharomyces* (288) in blue, *Rhodotorula* (218) in green, *Kluyveromyces* (146) in cyan, *Pichia* (135) in pink, *Debaryomyces* (113) in yellow, *Malassezia* (106) in grey. The remaining 188 genera with less than 85 ITS sequences are coloured using the distinctColorPalette function of the randomcoloR package. The coordinates of the sequences were generated using fMLC (Vu et al. 2018). The sequences are visualised using the rgl R package (https://r-forge.r-project.org/projects/rgl/)

As indicated above, sequence analyses of the D1/D2 domains of the LSU rRNA gene and the ITS regions of rDNA have been widely used for yeast identification as well as species delineation. Here we address the reliability and accuracy of these two barcode regions for the identification of yeasts using the barcode datasets generated at the Westerdijk Fungal Biodiversity Institute, Utrecht, the Netherlands, which consists of 4,356 ITS and 4,213 LSU sequences representing 80% of the yeast species present in the CBS collection (Fig. 2A–H). Vu et al. (2016) conducted such an analysis previously, allowing us to address the effect of some recent taxonomic revisions (e.g., Liu et al. 2015, Wang et al. 2015a, b). Based on the classification obtained at the time of the release of the barcode datasets in 2016, there were 1227 (1249) species, 184 (180) genera, 52 (52) families, 25 (21) orders, and 8 (11) classes present in the yeast ITS and LSU (in parentheses) datasets. Since 2016 340 (316) sequences of 17 (17) genera, including 135 (151) *Candida* and 56 (49) *Cryptococcus* sequences have been reassigned to other genera. To evaluate if these name changes resulted in an improvement for species and genus identification, we computed two metrics, a reasonable sequence similarity cut-off (optimal threshold) and its associated *F*-measure (Paccanaro et al. 2006, Vu et al. 2014) (Figs. 2E–H). The *F*-measure (having a value between 0 and 1) evaluates the confidence of a threshold used to assign a sequence to a species or genus which is computed by comparing the groupings obtained by clustering the sequences with the given threshold and with the groupings equivalent to the associated taxon names. This measure has been widely used for evaluating the results of clustering approaches and its formula can be found in Paccanaro et al. (2006) and Vu et al. (2014, 2016). The higher the value of the *F*-measure, the closer the grouping of sequences by sequence similarity to the grouping of the sequences based on taxon names. The optimal threshold has the highest *F*-measure for sequence identification. For the estimation of the optimal threshold for species identification, indistinguishable species that shared the same ITS and/or LSU sequences with the others, i.e., they were in the same group when clustering the datasets with a 100% identity score, were removed. The analysis shows the accuracy of identification of a random yeast sequence at different sequence similarity levels (threshold) and across the taxonomic ranks. The threshold value reflects the observed average similarity of sequences of members of the given taxonomic rank but does not consider a barcoding gap. The analysis is a good proxy for best-score sequence similarity comparisons, like those researchers routinely perform against GenBank and MycoBank sequence databases. At the species level, the best *F*-measures obtained in most of the datasets were high (~ 0.92), showing that except for the indistinguishable species (~ 5.3 and ~ 7.9% for ITS and LSU sequences, respectively), both ITS and D1/D2 LSU sequences had a strong identification power to identify yeasts. Within the *Basidiomycota*, a significant improvement for species identification was seen after the major changes in classification, as evidenced by the highest *F*-measure, which increased from 0.8 to 0.914 using ITS barcodes. The thresholds to predict species boundaries using ITS barcodes were 0.984 and 0.99 for *Ascomycota* and *Basidiomycota*, respectively, while they were 0.996 for both groups using the LSU barcode. Note that if the indistinguishable species were not excluded from the prediction, the obtained *F*-measures were < 0.82 and 0.81, although the thresholds for species boundaries were > 0.993 and 0.998 using ITS and LSU barcodes, respectively. At the genus level the obtained highest *F*-measures were low and varied from 0.56 to 0.83. Based on the *F*-measures obtained, ITS had a better identification power than LSU for yeast genera. In the *Basidiomycota*, an improvement was observed for genus identification in the updated taxonomy, as the best *F*-measure increased from 0.71 to 0.8 for ITS, and from 0.72 to 0.75 for LSU. For *Ascomycota,* the best *F*-measure increased from 0.6 to 0.68 for ITS, and from 0.56 to 0.58 for LSU. These low values of the *F*-measures obtained in the *Ascomycota* at the genus level, may suggest a need for further taxonomic improvement. This is likely due to the current circumscription of the polyphyletic genus *Candida*, which contains many species that are poised to be reassigned to other genera as more data become available (Fig. 3). Based on the whole datasets, the optimal thresholds predicted for genus identification using ITS (LSU) barcodes were 0.937 (0.989) (currently) and 0.939 (0.989) (2016 analysis). For the *Basidiomycota*, thresholds of 0.952 (0.989) (currently) and 0.958 (0.989) (2016 analysis) were obtained, and for the *Ascomycota*, 0.912 (0.992) (currently) and 0.940 (0.991) (2016 analysis).

Although the study comprised the largest collection of sequence data derived from authenticated collection cultures, the analysis does not suggest that sequence similarity cut-offs should be used as a sole criterion for the identification of yeast species and genera. Different clades may require different sequence similarity cut-offs, a reflection of different rates of substitution in various species or genera (Fig. 2A–D). Some genera show limited barcode sequence variation among species (e.g., *Debaryomyces, Cryptococcus*), while others are highly heterogeneous (e.g., *Clavispora, Metschnikowia*). A combination of the sequence similarity cut-off and its confidence is useful to evaluate the consequences of changes in classification upon the efficacy of barcode sequences in identifying yeast isolates. Overall, the recent revisions in the taxonomy of yeasts, especially those proposed for the *Basidiomycota*, resulted in an improvement in the ability of both barcode sequences to identify yeasts at the genus level.

Barcode sequences, especially ITS, are widely used to estimate fungal species richness in so-called metabarcoding studies of such diverse environments as soils, water, plants, and the human body (Findley et al. 2013; Tedersoo et al. 2014; Rämä et al. 2016; Botschuijver et al. 2017; Canini et al. 2019; Nilsson et al. 2019). ITS metabarcoding has also resulted in the recognition of many putative, yet undescribed new species collectively referred to as ‘fungal dark matter’ (Hibbett 2016; Lücking et al. 2020, 2021). A major debate is going on among mycologists if and how to publish formal descriptions of ‘dark matter’ species that are only documented from sequence data obtained from environmental DNA (Lücking et al. 2020, 2021), and whether environmental DNA can serve as type material (Hawksworth et al. 2016; Hongsanan et al. 2018; Zamora et al. 2018). Regardless of the outcome of this debate, DNA barcoding will remain a major tool, both in metabarcoding studies and in the identification of yeast isolates.

## Species, genomes and genomics

Recent innovations in genome sequencing techniques, coupled with advances in tools and computational powers for genome assembly and analyses, have led to a better understanding of the diversity, phylogeny, speciation, and genome evolution in the fungal kingdom. Fungal genomes undergo extensive genome reduction/expansion and rearrangements even between closely related species. Fungal genome sizes range from 2 Mb in *Microsporidia* to > 2 Gb in *Pucciniales* (Aime et al. 2017), and the total number of predicted protein-coding genes vary from a couple of thousands in *Microsporidia* to more than 35,000 in *Sphaerobolus* in the *Agaricomycotina* (Stajich 2017).

Whole-genome sequencing is rapidly becoming commonplace and can contribute greatly to a sound species recognition (James et al. 2020; Libkind et al. 2020). In addition to enlightening the evolution and genetics of the phenotype of the novel species, whole-genome data can provide a robust phylogeny, which can be of great utility not only to situate the species hierarchically, but also if the PSC is to be applied. Overall genome similarity metrics are useful in the application of the genetic species concept (GSC, Libkind et al. 2020). This will, however, require empirical validation. Having examined 36 well-delineated haplontic, heterothallic *Metschnikowia* species, Lachance et al. (2020) determined that an average nucleotide identity (ANI) value of 95% provides a good guideline to delineate species following the GSC, although the sharp boundary observed for prokaryotic microorganisms was not found in these yeast species. Whole genome analyses can resolve the problem of incongruent phylogenies obtained from different marker genes (Capella-Gutierrez et al. 2014). Improvements in high-throughput whole-genome sequencing, and the efforts of individual researchers and several large-scale initiatives such as the 1000 Fungal Genomes Project led by the United States Department of Energy Joint Genome Institute (https://mycocosm.jgi.doe.gov/mycocosm/home/1000-fungal-genomes) and the Broad Institute’s Fungal Genome Initiative (https://www.broadinstitute.org/fungal-genome-initiative/update-website-resources; Spatafora et al. 2017; Shen et al. 2018), have led to a major increase in the number of available fungal genomes. Fungal genome repositories such as FungiDB (https://fungidb.org/fungidb/app) have facilitated comparative genomics studies across the fungal kingdom (Stajich et al. 2011; Basenko et al. 2018) and phylogenomic analyses have allowed resolution of previously unresolved lineages, such as the *Ustilaginomycotina* (Kijpornyongpan et al. 2018) and the *Agaricomycetes* (Dentinger et al. 2016), that both include yeasts. Genome data can also allow inference of mating and life history strategies in cryptic fungi (Xu et al. 2007; Toome et al. 2014; Wu et al. 2015). Phylogenomic analyses supply ample markers for testing the GCPSR and provide a robust framework for the analysis of phenotypic evolution of fungi and the underlying genetic changes, including horizontal gene transfers, underlying such transitions. For example, it has been shown that the acquisition of genes required for anaerobic growth through horizontal gene transfer, followed by gene duplication and clustering, allowed *Saccharomyces* to become an ideal brewing yeast (Gojković et al. 2004; Piškur et al. 2006).

The increased availability of novel sequencing technologies, including single-cell sequencing and broad sequencing of environmental samples (e.g., metagenomics), promises to reveal even more genotypic and ecological diversity and complexity in the fungal kingdom (Gabaldón 2020c; Rosling et al. 2011; James et al. 2013; Ahrendt et al. 2018). In recent years, advancements in long-read sequencing techniques, such as PacBio and Oxford Nanopore MinION, allow the assembly of complete fungal genomes at the chromosome level, as they are able to generate sequencing reads that are long enough to span genomic regions that are highly repetitive (e.g., rDNA) or enriched with transposon repeats (e.g. centromeres), which are difficult to assemble from short reads generated by sequencing platforms such as Illumina. Improvements in genome assemblies from long-read sequencing has also facilitated better allele phasing (assignment of alleles to their respective chromosome) in diploid species or diploid hybrids of haploid species, and thus, more robust analyses of mitotic recombination and their effects on fitness and evolution of the hosts.

Complete genome data, including full-length chromosomes sequences and the best possible gene annotation is necessary to understand many evolutionary processes, such as gene duplication, mutations and acquisition of novel genetic material that underlie yeast evolutionary genomics (Dujon 2010; Dujon and Louis 2017). Complete genome assemblies enable more robust analyses of genome rearrangements such as inversions and translocations, which have shown that: (1) such chromosomal changes occur frequently between closely related species, even among isolates of the same species; (2) many of these chromosomal rearrangements were mediated by transposable elements, and in some cases by chromosomal regions (such as centromeres) that are enriched with transposons and their remnants; and (3) chromosomal rearrangements alone are sufficient to establish reproductive barriers, and could have played significant roles in fungal speciation (Janbon et al. 2014; Sun et al. 2017; Yadav et al. 2020). Together with genetic and molecular biology studies, analyses of complete fungal genomes also revealed that fungal centromeres are highly diverse, ranging in size from 125 bp in *S. cerevisiae*, to a few kilobases in certain *Candida* species, to tens or even hundreds of kilobases in some *Cryptococcus* species that harbour large regional centromeres (Guin et al. 2020). Fungal centromeres also differ significantly in their structures, varying from well-defined point centromeres to short regional centromeres with terminal repeats, to large regional centromeres enriched with transposons and their remnants. While regional centromeres are likely defined epigenetically, centromere-specific sequence motifs may still play critical roles in centromere characterization and function (Yadav et al. 2018; Navarro-Mendoza et al. 2019; Guin et al. 2020; Sankaranarayanan et al. 2020).

As sequencing prices drop, comparative genomics studies can address genomic diversity within single species, or even populations. Population genomics studies have revealed that fungal species are genetically heterogeneous. This diversity extends well beyond polymorphisms of shared loci, also eliciting differences in the presence and absence of genes among genomes from strain to strains. Although only a limited number of model fungal species have been explored in this regard, it seems that the concept of pan-genome, developed for bacteria, also applies to fungi (Naranjo-Ortiz and Gabaldón 2020). This large genetic diversity within fungal species as well as the realization that non-vertical modes of evolution occur in fungi, challenges the identification and separation of species based on the basis of specific marker loci (Lücking et al. 2020). The selection of barcoding genes heavily relies on historical choices and not on the empirical testing of the ability of each locus to properly differentiate strains from different species. As different genes may show different levels of conservation in different clades, the very concept of whether there is a universal fungal barcoding gene remains a matter of debate. In this context, the continuous developments in sequencing and computing technologies coupled with more affordable and user-friendly platforms may soon lead to a situation in which low coverage sequencing and comparison with extensive databases become the cost-effective solution for the accurate delineation of a fungal species (Gabaldón 2020b). However, for this to be a reality several issues remain to be resolved, namely establishing standardized pipelines, curating errors from public databases, and superimposing the boundaries of our current morphology-based species definition to the expanding knowledge of genomic divergence (Stavrou et al. 2018; Xu 2020).

## The species description, best practices

As stated by Mayden (2002), “species descriptions represent testable hypotheses, just the same as what is presented in other areas of the natural sciences.” The mere isolation of a contaminant whose DNA-barcode sequence differs by four substitutions from an existing species hardly justifies the formal description of a novel species. Whether or not the observed differences are sufficient to erect a new species must be viewed in the context of a species concept. An overview of species concepts used in mycology is provided by Lücking et al. (2020) and those frequently used for yeasts are discussed in more detail here (see Table 1). The choice of a species concept determines the characters and tools used for its delineation and identification. For example, the demonstration of reciprocal monophyly required by the PSC makes no sense if the extent of sequence polymorphism is not known because a species is represented by only a single isolate. Application of the criterion of genealogical concordance, which combines the PSC with the BSC requires examination of several individuals. Once the existence of a novel species is demonstrated from its genealogy (PSC/GCPSR), reproduction (BSC), and phenotype (PhenSC), efforts should be made to identify the habitat, that is, where the species occurs naturally, and its niche. This entails repeating the isolation from similar substrates, using a sound sampling strategy that will help identify the actual habitat, or search for its presence in metabarcoding libraries.

Some researchers advance that naming of yeast species should be delayed until all taxa within a phylogenetic grouping have been identified (see e.g., Kwon-Chung et al. 2017). The reality is that there will be a near continual recognition of new taxa as science advances. Once a species is described formally it can be thoroughly tested as new specimens are identified (Fig. 1). A striking example is *Candida auris*, which was described from only a single isolate obtained from the ear of an inpatient in Japan (Satoh et al. 2009). Within one decade after its discovery *C. auris* has become one of the major global fungal pathogens (Lockhart et al. 2017a, b; de Jong and Hagen 2019).

A scrupulous scrutiny of yeast species descriptions published in the past might well reveal that a substantial proportion would not meet modern best practices (Yurkov et al. 2021). Perhaps one of the most important aspects of describing new taxa should be a sensitivity to the user community. Descriptions should be informative and founded on the highest quality biology and provide enough detail for unambiguous identification (Aime et al. 2021). In an editorial published in the journal ‘*Yeast’*, Lachance (2020) listed a number of essential requirements, namely high-quality work, reference to the relevant literature including the latest edition of ‘*The Yeasts, a Taxonomic Study’* (Kurtzman et al. 2011a) or its electronic successor https://theyeasts.org, expert etymology, a standard list of growth properties, high-quality photographs of all stages of the life cycle, evidence of due diligence in observing sexual reproduction, deposition of barcode sequences (e.g. ITS and D1/D2 domains of the LSU rRNA gene), a lucid statement of applicable species concepts, a sequence-based tree appropriate to the species concept invoked, and clear information on the geography and habitats of isolates. In addition to these essential features, the editorial recommended that the proposal of a new species be documented from distinct, multiple isolates, in the absence of which a strong justification should be given to warrant publication of a description. Additionally, deposition and annotation of a (draft) genome were considered desirable, and authors were encouraged to couch the description in an ecologically meaningful framework. Karyotype information was also recommended. These and other recommendations have been taken up by the *International Commission on the Taxonomy of Fungi*, and formalized in a publication that includes model manuscripts, a checklist, and templates for descriptions (Aime et al. 2021). Taken together, these requirements ensure that a new species is thoroughly characterised and distinct from other species, that it can be identified by other researchers, and that its reference material is available for further research.

Yeast morphology has yielded the way to nutritional tests and later sequence information for the purpose of delineation and identification of species. It remains that morphology contributes a large proportion of what we regard as representative of a yeast species. Microscopes are unlikely to improve much in the foreseeable future and so the onus is on individual systematists to choose the most appropriate approaches. The ability to generate good images is based almost entirely on adequate training. Characteristics such as cell wall composition, nucleotide ratios, principal ubiquinone isoprenoid length have had their historical importance, but it is fair to question their current relevance. Growth tests formulated primarily by L.J. Wickerham provide much insight on the adaptation of yeast species to their environment but are laborious and fraught with much response variation between laboratories. Most of the commercially available kits used for identification of yeast isolates can also be used to perform growth tests, but they do not follow the traditional standard method, i.e., Wickerham’s tube method or auxanograms, and lack validation against time-tested methods. As the kits focus on the identification of a limited number of clinical and food-borne yeasts, their results are often less informative for species that are not normally associated with those substrates. For example, the multi-drug resistant emerging pathogen, *Candida auris* (see also above), has been repeatedly misidentified by commercial kits (Lockhart et al. 2017b; Mizusawa et al. 2017). Although specialized identification with some of these test kits has improved (Arastehfar et al. 2019), their use in a clinical setting is rapidly being displaced by MALDI-TOF and PCR or sequence-based tests. Effort is needed to find better ways to capture growth responses. It is difficult to imagine how one can describe a yeast species without such data, as growth responses are a window on the fundamental niche of a yeast species and thus represent valuable information relevant to ecology. Examples are yeasts found in sugar-rich substrates (Péter et al. 2017), soil yeasts able to assimilate the by-products of cellulose and lignin degradation (Botha 2011), or the need for yeasts that are pathogenic to mammals, including humans to grow above 35 °C (Robert et al. 2015).

## Naming of species and the rules of nomenclature

There are three general ways in which fungal species have been named. The first one is in honour or homage to a distinguished scientist, such the basidiomycete genus *Kwoniella*, named in honour of Dr. June Kwon-Chung. Second, species are named in recognition of a location associated with their discovery, such as *Kwoniella botswanensis*, discovered from Mopane Trees in Botswana. Third, species are named after an attribute, such as the substrate on which they were found as in the case of *Cryptococcus floricola*, isolated from the nectar of flowers on the Canary Islands. Given the vast number of fungi that remain to be discovered and named creativity in the generation of new names will be an asset. A consensus among fungal biologists is lacking on what approach to naming is most desirable. For example, yeasts named after a locality may later be found to be more widespread, and the same holds for the source of isolation.

Two aspects are important when formally describing any new species including yeasts. The first is taxonomy, which includes the characters used to differentiate species. Although no formal requirements exist for good taxonomy, community standards and best practices, such as those outlined in the sections above, exist to guide authors and journals in the publication of new taxa that meet rigorous scientific standards as well as the needs of the user community. The other realm is that of nomenclature, which concerns the legal requirements that dictate how to publish and name a new taxon. In other words, while taxonomy is concerned with what constitutes a species and how to describe it, nomenclature concerns how to name a species, regardless of how it has been defined.

Nomenclatural rules applicable to yeasts are found in the ‘*International Code of Nomenclature for algae, fungi, and plants’* (ICNafp, or the *Code*) (Turland et al. 2018; https://www.iapt-taxon.org/nomen/main.php). The ICNafp is updated every six years, and the requirements in each new code supersede those of prior codes. For publication of a new name to be *Code*-compliant it must fulfil the rules for: (1) effective publication, in other words that it be published in a sanctioned journal, explained in detail in Arts. 29–31; (2) valid publication, which primarily deals with correct designation of holotypes or basionyms, which are explained in detail in Arts. 32–45; and (3) legitimate publication, which broadly concerns the usage of names based on previously published taxa as well as publication of superfluous names, as detailed in Art. 6. Any names that do not meet the requirements for all three are unavailable for use. Illegitimate names may or may not also be invalid. General guidelines for how to avoid publishing invalid or illegitimate names are provided in Aime et al. (2021); more detailed guidelines for yeast taxa follow.

The most common errors in erecting new names for yeasts species are: (1) improper designation of the holotype and holotype institution (Arts. 8.4 and 40.7). Most commonly, this is the failure to designate a *single* institution in which the holotype resides; (2) not designating a metabolically inactive specimen as the holotype. To fulfil the latter requirement of the *Code*, either a dried specimen or a metabolically inactive culture should be prepared, designated and deposited in a repository. This can be achieved by drying a culture plate and preserving it in a fungarium/herbarium or to deposit the culture in a culture collection that preserves holotypes as lyophilized samples or cryopreserved at ultra-low temperatures. As of January 1, 2019, the words “metabolically inactive” need to be included along with the institution designation in the holotype designation to show this clearly (Arts. 40.7 and 40.8).

In the ICNafp, a holotype is a single collection, specimen, or isolate that serves as the reference for its name for all contemporary and future researchers. A holotype should not only be selected as the best representative of a name but should also be deposited in an internationally recognized and easily accessible biorepository (fungarium, herbarium, or culture collection), and wherever possible, accompanied by an isotype (metabolically inactive duplicates of the holotype). When a researcher deposits subcultures of a strain that was selected to represent a nomenclatural type of a name in two (or more) culture collections, and if all of these deposits are preserved in a metabolically inactive state, only one can be designated as a metabolically inactive holotype and other deposits should be indicated as isotypes. The ICNafp recommends the deposition of living cultures prepared from the holotype in at least two culture collections (Rec. 8B1). These cultures are designated as ex-types, i.e., derived from the metabolically inactive holotype.

While there are no formal requirements for depositing viable cultures, it goes without saying that ex-type cultures should be deposited in publicly accessible international collections and be available to the scientific community, a list of which are available through the *Culture Collections Information Worldwide* of the World Federation for Culture Collections and World Data Center for Microorganisms (http://www.wfcc.info). It is preferable that duplicates of the type material be deposited in more than one country.

Fortunately, invalid names can be later validated by correcting the errors in a subsequent publication. For example, a new name that was published invalidly because several cultures were listed without indicating which was the holotype, can be validated in a later publication by selection and designation of one of the cultures to serve as holotype (as long as it is preserved in a metabolically inactive state). In these cases, the original author names are followed by “ex” and the names of the authors who validated the name. Illegitimate names can, in rare cases, be nominated for conservation, but this is rarely done, and illegitimately published names of distinct taxa are usually corrected by publication of entirely new names for the replaced name. In these cases, the new name is followed by the authors of the new name and the designation “nom. nov.”, and the basionym is cited as the original name and publication. In cases where names were both invalidly and illegitimately published, a correct holotype designation must also be included in the nom. nov. description. Other formal requirements for valid and legitimate publication of names are explained in May et al. (2019) and Turland (2019).

The ICNafp provides indications on how to name hybrids between defined species (Turland et al. 2018). However, these indications allow enough room for alternative choices. For instance, a hybrid taxon can be given a standard species name, without any indication of the hybrid nature in the name. This is the case of many naturally occurring hybrids that were described before their hybrid nature was discovered. For instance, *S. pastorianus* (Vaughan Martini and Martini 1987). Hybrids between two or more described taxa can also be named using the multiplication sign “×”, which is placed before the name of an intergeneric hybrid or before the epithet in the name of an inter-specific hybrid. This is usually the choice for artificially created hybrids in which both parental species are known, e.g., *S. cerevisiae* × *S. uvarum*. Nguyen and Boekhout (2017) provided some guidelines for naming yeast hybrids that can also be used for other hybrid yeasts. The species that comprise the hybrid are given in alphabetical order, e.g., strain CBS 8615 (CID1) is a *S. cerevisiae* × *S. kudriavzevii* × *S. uvarum* hybrid, and if the ploidy of the parent species is known this is given after each species name. For example, *S. bayanus* CBS 380^T^ is *S. cerevisiae* ˂1% × *S. eubayanus* 37% × *S. uvarum* 63%. Note that different hybrids may differ in their evolutionary trajectories and a distinction is to be made between recently formed hybrids and those that have evolved over a long history that even may represent species (see discussion above).

Additionally, the use of the prefix “notho-” (optionally abbreviated “n-”) added to the term denoting the rank of the taxon can be used to emphasize the hybrid nature of a taxon, although this convention is not commonly used in yeasts. Of note, the prefix notho- and the multiplication sign are not part of the actual species name and should be disregarded for nomenclatural purposes, such as synonymy or homonymy. Furthermore, the designation as nothotaxon of any taxon that is believed to be of hybrid origins is optional, and a nothotaxon cannot be designated unless at least one parental taxon is known or can be postulated, but this leaves ample room to consider different possible ways of postulating a parental taxon.

## Conclusions and perspectives

From the above it is clear that many species concepts have been used over time to describe yeast diversity. Initially a PhenSC was used, mainly taking into consideration cellular and colony morphologies and growth responses. Mating experiments with or without a genetic analysis of the offspring were used to assess the presence of reproductive barriers important to our understanding of species boundaries under the BSC. However, the increasing number of yeast hybrids observed indicate that the application of the BSC is not without risk and must be based on a thorough genetic analysis. The use of (multiple) molecular markers, such as sequences of the D1/D2 domains of the LSU rRNA gene, the ITS rDNA region, and other (protein coding) sequences, as proxies for species delimitation, and comparison with species boundaries set under the BSC has yielded insights into thresholds of nucleotide divergence values that may be considered to set species hypotheses under the GSC. The phylogenetic grouping of yeast isolates using partial or entire genome DNA sequences under the PSC/GCPSR has recently gained in popularity. Finally, unknown species identified by ITS metabarcoding from environmental DNA, usually referred to as ‘fungal dark matter’ (Lücking et al. 2020, 2021), need attention as well. We believe that the species hypotheses originating from the above approaches will need thorough experimental testing, using genetics, ecology, and comparative and functional genomics approaches that will take (several or many) experimental iterations (Fig. 1). Note that testing of species hypotheses usually starts once these have been postulated, e.g., in a formal species description (Nguyen and Gaillardin 2005; Louis 2009; Büker et al. 2013; Pérez-Través et al. 2014; Pryzcz et al. 2014, 2015; Hagen et al., 2015, 2017; Marcet-Houben and Gabaldón 2015; Kwon-Chung et al. 2017; Nguyen and Boekhout 2017; Passer et al. 2019: Priest et al. 2021). This process of testing species hypotheses has started with a few model species, such as *S. cerevisiae*, but also the *Cr. neoformans* species complex and *M. lychnidis*-*dioicae* (Fig. 1), and this will be expanded to other species complexes in the future. To gain better insight in the genomic dynamics of evolving species we strongly recommend analysing completed rather than draft genomes. Only detailed knowledge of yeast genome organization, for isolates and their possible offspring—including structural genetic elements, such as chromosomes, centromeres, genes/open reading frames, exons/introns, repeated sequences, non-coding RNAs including tRNAs, rRNAs, snoRNAs, microRNAs, and transposons—will give us insight into the evolution of the genomes within and betweens species, how this affects selective advantage, and the data for implementation of DFSC. Such analyses may include aspects of structural biology, such as the three-dimensional structure of proteins of, e.g., pheromone peptides, pheromone receptors, and transcriptomics studies of various mating systems and physiology, hybridization, chromosome evolution, and the meiosis-related cell machinery, to better understand mechanistic aspects of pre- and postzygotic isolation that determine gene flow between populations. Also (in)compatibility between nuclear and mitochondrial genomes in such processes can be considered. Eventually, this will result in a more accurate, ‘holistic’ species concept that also will take into consideration intrinsic complexities, e.g., due to hybridization and introgression, forces that illustrate, reflect, and reinforce species boundaries but continue to confuse many investigators into considering (and even inaccurately asserting and espousing) the misconception that species boundaries are artificial edifices. Such a species concept likely will take the form of (small) speciation networks that relate to other such networks, e.g., via introgression and hybridization processes, and that address the genetic cohesiveness of species complexes more fully and accurately than the current tree-like models. Given the rapid developments of omics and bioinformatics technologies, the future will hold many surprises with respect to our understanding of the (fuzzy) boundaries between yeast species.
